# A matched-pair analysis on survival and response rates between German and non-German cancer patients treated at a Comprehensive Cancer Center

**DOI:** 10.1186/s12885-019-6241-9

**Published:** 2019-10-30

**Authors:** Marie K. Budde, Walther Kuhn, Mignon-Denise Keyver-Paik, Friedrich Bootz, Jörg C. Kalff, Stefan C. Müller, Thomas Bieber, Peter Brossart, Hartmut Vatter, Ulrich Herrlinger, Dieter C. Wirtz, Hans H. Schild, Glen Kristiansen, Thorsten Pietsch, Stefan Aretz, Franziska Geiser, Lukas Radbruch, Rudolf H. Reich, Christian P. Strassburg, Dirk Skowasch, Markus Essler, Nicole Ernstmann, Jennifer Landsberg, Benjamin Funke, Ingo G. H. Schmidt-Wolf

**Affiliations:** 1 Department of Integrated Oncology, Center for Integrated Oncology (CIO), University Hospital Bonn, Venusberg-Campus 1, 53127 Bonn, Germany; 2 Department of Gynecology and Obstetrics, University Hospital Bonn, Bonn, Germany; 3 Department of Otorhinolaryngology, University Hospital of Bonn, Bonn, Germany; 4 Department of Surgery, University Hospital of Bonn, Bonn, Germany; 5 Department of Urology, University Hospital Bonn, Bonn, Germany; 6 Department of Dermatology and Allergy, University Hospital Bonn, Bonn, Germany; 7 Department of Internal Medicine III, University Hospital Bonn, Bonn, Germany; 8 Department of Neurosurgery, University Hospital Bonn, Bonn, Germany; 9 Department of Neurology, University Hospital Bonn, Bonn, Germany; 10 Department of Orthopedic and Trauma Surgery, University Hospital Bonn, Bonn, Germany; 11 Department of Radiology, University Hospital Bonn, Bonn, Germany; 12 Institute of Pathology, University Hospital Bonn, Bonn, Germany; 13 Department of Neuropathology, University Hospital Bonn, Bonn, Germany; 14 Institute of Human Genetics, University Hospital Bonn, Bonn, Germany; 15 Institute of Psychosomatic Medicine and Psychotherapy, University Hospital Bonn, Bonn, Germany; 16 Department of Palliative Medicine, University Hospital Bonn, Bonn, Germany; 17 Department of Oral and Maxillofacial Plastic Surgery, University Hospital Bonn, Bonn, Germany; 18 Department of Internal Medicine I, University Hospital Bonn, Bonn, Germany; 19 Department of Internal Medicine II, University Hospital Bonn, Bonn, Germany; 20 Department of Nuclear Medicine, University Hospital Bonn, Bonn, Germany; 21 Center for Health Communication and Health Services Research, Department of Psychosomatic Medicine and Psychotherapy, University Hospital Bonn, Bonn, Germany

**Keywords:** Migrants, Cancer, Survival, Inequalities, Matched pair analysis

## Abstract

**Background:**

Research shows disparities in cancer outcomes by ethnicity or socio-economic status. Therefore, it is the aim of our study to perform a matched-pair analysis which compares the outcome of German and non-German (in the following described as ‘foreign’) cancer patients being treated at the Center for Integrated Oncology (CIO) Köln Bonn at the University Hospital of Bonn between January 2010 and June 2016.

**Methods:**

During this time, 6314 well-documented patients received a diagnosis of cancer. Out of these patients, 219 patients with foreign nationality could be matched to German patients based on diagnostic and demographic criteria and were included in the study. All of these 438 patients were well characterized concerning survival data (Overall survival, Progression-free survival and Time to progression) and response to treatment.

**Results:**

No significant differences regarding the patients’ survival and response rates were seen when all German and foreign patients were compared. A subgroup analysis of German and foreign patients with head and neck cancer revealed a significantly longer progression-free survival for the German patients. Differences in response to treatment could not be found in this subgroup analysis.

**Conclusions:**

In summary, no major differences in survival and response rates of German and foreign cancer patients were revealed in this study. Nevertheless, the differences in progression-free survival, which could be found in the subgroup analysis of patients with head and neck cancer, should lead to further research, especially evaluating the role of infectious diseases like human papillomavirus (HPV) and Epstein-Barr virus (EBV) on carcinogenesis and disease progression.

## Background

In 2016, the number of foreigners in Germany reached 10.04 million [1] and the number of asylum-seeking people reached the highest level since 1953 (745.545) [2].

Thus, the task of providing appropriate health care to foreign cancer patients has gained major importance. Prevention and treatment programs should not only be reachable for German but also for foreign patients at the same extent.

It is known that there are differences in access to health care between groups with different socioeconomic status [3] and that socioeconomic deprivation is associated with a poor prognosis for several cancer entities, even in highly developed countries like Germany [4–6]. However, to our knowledge, the prognostic value of nationality on survival has not been investigated yet. It is still not known if nationality is an independent prognostic factor among patients with cancer disease in Germany. The possible revealing of differences may help to create a fairer health care system taking disadvantaged groups more into account. Furthermore, knowledge about differences in the outcome of German and foreign patients may allow a better understanding of cancer etiology and biological factors [7].

Given these dynamics, the aim of this matched-pair analysis is to examine whether differences in survival and response rates of German and foreign cancer patients being treated at the University Hospital of Bonn exist.

## Methods

### Patients

Between January 2010 and June 2016, 6314 cancer patients were collected in a cancer register of the CIO at the University Hospital of Bonn.

**Fig. 1:**
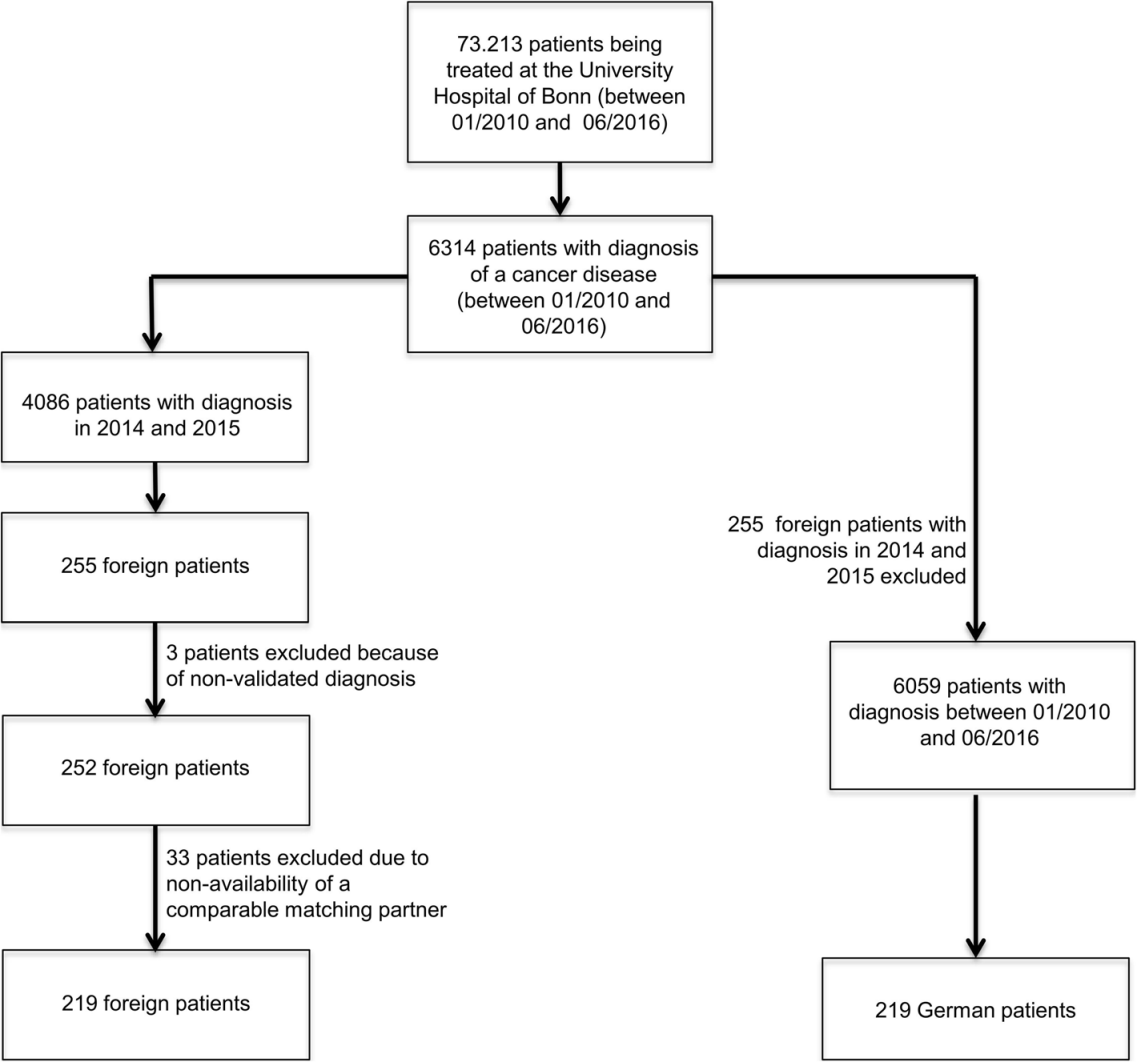
Strategy of data collection and matching process**.** In the group of patients with diagnosis in 2014 and 2015, nationality has been examined. 255 foreign patients could be found. To enhance the possibility of finding an appropriate matching partner, the period of time in which we searched for the German matching partner was preset from 01/2010 to 06/2016 (however, we tried to ensure a concordant length of follow-up by looking for a matching partner with same year of diagnosis first). After excluding three patients due to non-validated diagnosis und 33 patients due to non-availability of a comparable matching partner, 219 foreign patients remained who could be matched to compatible German patients

To find cancer patients of non – German nationality only patients diagnosed in 2014 and 2015 were examined. 255 out of 4086 cancer patients were foreign and included in this study. Three of them had to be excluded due to insufficient clinical data and not-validated diagnoses. Figure 1 shows further details about the strategy of data collection.

### Matched pair-analysis

Each foreign patient was matched with one German control patient in a fashion blinded to patients’ outcomes. The criteria for the matching process were defined as follows: Diagnosis (based on ICD-10 and ICD-O-3), disease status (primary case vs. recurrence), tumor stage (UICC status for solid tumors, Ann-Arbor status for lymphomas, Durie and Salmon status for multiple myelomas and Binet status for CLL), sex and age (±10 years). Additionally, Gleason score was used to find a matching partner for patients with prostate cancer and Clark Level was used to find a matching partner for patients with malignant melanoma. The estrogen, progesterone and erbB2 receptor stage was supplementary employed to find a matching partner for patients with breast cancer. It was tried to find German matching partner who received their diagnosis in 2014 and 2015. If no appropriate matching partner with day of first diagnosis in 2014 and 2015 could be found, the period of time, in which the matching fashion was performed, was extended to the period of time between January 2010 and June 2016.

Thirty-three foreign patients had to be excluded due to non-availability of a comparable matching partner.

Finally, 219 foreign cancer patients could be matched to 219 German cancer patients. Details of patients’ characteristics and their distribution among both groups are shown in Table 1.

**Table 1:** Distribution of patients’ characteristics among both groups (*n* = 438)

All patients (*n* = 438)			Foreigners (*n* = 219)		German (*n* = 219)			
			n	%	n	%	*p*-value	chi-square
Age (years)								
< 60			127	58.0	114	52.1		
≥ 60			92	42.0	105	47.9		0.211
Median			55.1		58.6		0.01	
Range			Min. = 20 Max. = 93		Min. = 24 Max. = 94			
Sex								
Male			123	56.2	124	56.6		
Female			96	43.8	95	43.4		0.923
Follow-up (months)								
Median			14.5		17.9		0.001	
Range			Min. = 0.1 Max. = 34.5		Min. = 0.3 Max. = 73.8			
All patients with solid tumors (*n* = 382)			Foreigners (*n* = 141)		German (*n* = 141)			
			n	%	n	%		
UICC								
0			2	1.4	2	1.4		0.979
I			28	19.9	25	17.7		
II			12	8.5	13	9.2		
III			16	11.3	20	14.2		
IV			36	25.5	37	26.2		
X			47	33.3	44	31.2		
Total			141	100.0	141	100.0		
WHO brain								
I			0	0	0	0		1.000
II			1	7.7	1	7.7		
III			2	15.4	2	15.4		
IV			10	76.9	10	76.9		
Total			13	100.0	13	100.0		
FIGO								
DCIS			3	8.1	3	8.1		0.998
I			7	18.9	8	21.6		
II			6	16.2	4	10.8		
III			3	8.1	3	8.1		
IV			4	10.8	4	10.8		
X			14	37.8	15	40.5		
Total			37	100.0	37	100.0		
Additionally Scores								
Gleason								
6			1	10.0	1	10.0		0.878
7			3	30.0	3	30.0		
8			3	30.0	2	20.0		
9			3	30.0	3	30.0		
X			0	0.0	1	10.0		
Total			10	100.0	10	100.0		
Clark Level								
I			0	0.0	0	0.0		0.315
II			0	0.0	0	0.0		
III			2	25.0	3	37.5		
IV			4	50.0	5	62.5		
X			2	25.0	0	0		
All patients with malignant hematological diseases (*n* = 56)								
			Foreigners (*n* = 28)		German (*n* = 28)			
			n	%	n	%		
Lymphoma			13	46.4	13	46.4		1.000
Leukemia			8	28.6	8	28.6		
Multiple Myeloma			7	25.0	7	25.0		
Ann Arbor								
I			1	7.7	2	15.4		0.829
II			1	7.7	2	15.4		
III			0	0.0	0	0.0		
IV			7	53.8	6	46.2		
X			4	30.8	3	23.1		
Total			13	100.0	13	100.0		
Durie and Salmon								
I			0	0.0	0	0.0		0.135
II			1	14.3	1	14.3		
III			3	42.9	6	85.7		
X			3	42.9	0	0		
Total			7	100.0	7	100.0		
Binet								
A			2	100.0	2	100.0		1.000
B			0	0.0	0	0.0		
C			0	0.0	0	0.0		
X			0	0.0	0	0.0		

The characteristics of the two groups after matching were widely balanced but significant differences still existed regarding patients’ age. Additionally, matching partner with the same treatment could only be found for 138 foreign patients (63.0%). In the rest of the cases, the type of treatment differed. Unless otherwise stated, we accepted the differences in therapy in our calculations, as we were able to ensure the accordance of diagnoses and tumor stages (Table 1; Table 2). The differences in therapy can be divided into the following: the absence of radiation or adjuvant chemotherapy after surgery, the absence of surgery to reduce the tumor size in palliative situations, the absence of a stem cell transplantation and the absence of a immune or hormone therapy in one patient compared to his/her matching partner. Furthermore, differences in immune or hormone therapy regimes, chemotherapy protocols, the use of supplementary therapies or the complete type of treatment must be mentioned.

**Table 2:** Distribution of the different tumor entities in the complete cohort (*n* = 438)

Tumor entities	n	%
gynecological cancer	74	16.9
breast	44	10.0
cervix uteri	10	2.3
ovary	10	2.3
DCIS	6	1.4
corpus uteri	4	0.9
head and neck	42	9.6
larynx	16	3.7
oropharynx	6	1.4
palate	4	0.9
hypopharynx	4	0.9
parotid gland	4	0.9
other illdefined sites in the lip, oral cavity and pharynx	2	0.5
nasopharynx	2	0.5
floor of mouth	2	0.5
accessory sinuses	2	0.5
colorectal and anal cancer	38	8.7
colon	32	7.3
rectum	4	0.9
anal	2	0.5
urological cancer	36	8.2
kidney (except renal pelvis)	18	4.1
bladder	16	3.7
renal pelvis	2	0.9
thyroid gland	30	6.8
non-melanoma skin cancer	26	5.9
basal cell cancer	20	4.6
squamous cell cancer	4	0.9
Bowen disease	2	0.5
lymphoma	26	5.9
non-follicular lymphoma	20	4.6
Hodgkin lymphoma	4	0.9
other specified types of T−/NK-cell-lymphoma (C83)	2	0.5
brain and spinal cord	26	5.9
glioblastoma	20	4.6
oligoastrozytoma	2	0.5
oligodendroglioma	2	0.5
ependymom	2	0.5
male genital tract	24	5.5
prostate	20	4.6
testis	4	0.9
pancreas	20	4.6
adeno carcinoma	16	3.7
NET	4	0.9
leukemia	16	3.7
AML	10	2.3
CLL	4	0.9
CML	2	0.5
melanoma	16	3.7
nodular	6	1.4
superficial spreading	6	1.4
acral lentiginous	2	0.5
without further indication	2	0.5
stomach	14	3.2
adeno carcinoma	12	2.7
NET	2	0.5
multiple myeloma	14	3.2
liver and biliary tracts	12	2.7
bronchus and lung	8	1.8
SCLC	4	0.9
NSCLC	2	0.5
NET	2	0.5
sarcomas	6	1.4
chondrosarcoma	4	0.9
synovial sarcoma	2	0.5
adrenal gland	4	0.9
esophagus	2	0.5
CUPs	2	0.5
Kaposi sarcoma	2	0.5
Total	438	100

### Statistical analysis

The software IBM SPSS (Chicago, IL) statistics for Mac (version 23) was used for statistical analysis. To compare nominal and ordinal matching variables of German and foreign cancer patients Pearson’s Chi-Square test was assessed. Student’s *t* test was used to compare ages and follow-up times between groups. All tests were two-sided and *p* < 0.05 was preset as the cutoff for significance.

Survival analysis for both groups was performed using Kaplan-Meier analysis (log rank test).

Overall survival (OS) was defined from the day of diagnosis until death. Progression-free survival (PFS) was defined from the day of diagnosis until disease progression or death by any cause. Time to progression (TTP) was defined from the day of diagnosis until disease progression or death related to cancer disease.

Response criteria followed the Response Evaluation Criteria In Solid Tumors (RECIST) and were subdivided into complete remission (CR), partial remission (PR), stable disease (SD) and progressive disease (PD).

Response criteria for hematological cancer diseases were adapted to the Response evaluation criteria in solid tumors (RECIST criteria). ‘Major molecular response’ and ‘cytogenetic response without major molecular response’ in chronic myeloid leukemia were used as ‘CR’ and ‘PR’ in statistical analysis. ‘VGPR’ which occurred in three cases of multiple myeloma was considered to be ‘PR’.

## Results

### Patients’ characteristics

#### Foreign patients

The mean age of the foreign cohort (*n* = 219) was 55.1 (range 20–93). One hundred twenty-seven patients (58.0%) were younger than 60 years and 92 patients (42.0%) were at least 60 years old. One hundred twenty-three patients (56.2%) were male and 96 (43.8%) were female. Follow-up data was available in 217 cases (99.1%) within the group. The mean follow-up time was 14.5 months ranging between 0.1 and 34.5 months. One hundred forty-one foreign patients (64.4%) had a national health insurance and 78 foreign patients (35.6%) had a private health insurance.

#### German patients

The mean age of the German cohort (n = 219) was 58.6 (range 24–94). One hundred fourteen of the German patients (52.1%) were younger than 60 years and 105 patients (47.9%) were at least 60 years old. One hundred twenty-four of them were men (56.6%) and 95 were women (43.3%). Follow-up data was available in all cases with a mean follow-up time of 17.9 months ranging between 0.3 and 73.8 months. One hundred seventy-five German patients (79.9%) had a national health insurance and 44 foreign patients (20.1%) had a private health insurance.

### Matched pairs’ characteristics

Two hundred nine matched pairs (95.4%) were cases with a primary tumor and 10 matched pairs (4.6%) were firstly seen with recurrences. One hundred ninety-one cases of the matched pairs (87.2%) had a solid tumor and 28 (12.8%) had a hematological malignant disease.

The largest group of cancer was of gynecological origin (37 pairs, 16.9%) including breast, ovarian, cervical and endometrial cancer.

Details of all entities included in this study are shown in Table 2.

### Foreign patients’ nationalities

The most common nationality was Turkish (29 patients, 13.2% of all foreign patients).

Russian (18 patients, 8.2%) was the second leading nationality in the foreign patients’ cohort. Italian nationality was found in 14 cases (6.4%). Patients with a nationality described as “Arabic” and patients from the United Arab Emirates each occurred in 12 cases (5.5%).

The distribution of the foreign patients’ nationalities is shown in Table 3.

**Table 3:** Distribution of the foreign patients’ nationalities

	n	% Subset	% Total
Eastern Europe			
Russian	18	29.0	8.2
Polish	9	14.5	4.1
Romanian	6	9.7	2.7
Croatian	5	8.1	2.3
Ukrainian	5	8.1	2.3
Serbian	4	6.5	1.8
Bulgarian	3	4.8	1.4
Bosnian	2	3.2	0.9
Czech	2	3.2	0.9
Latvian	2	3.2	0.9
Yugoslavian	1	1.6	0.5
Albanian	1	1.6	0.5
Hungarian	1	1.6	0.5
Macedonian	1	1.6	0.5
Moldavian	1	1.6	0.5
Kazakh	1	1.6	0.5
Total	62	100.0	28.3
Southern Europe/Turkey			
Turkish	29	50.9	13.2
Italian	14	24.6	6.4
Greek	7	12.3	3.2
Spanish	5	8.8	2.3
Portuguese	2	3.5	0.9
Total	57	100.0	26.0
Middle East			
United Arab Emirates	12	22.2	5.5
Arabic	12	22.2	5.5
Syrian	9	16.7	4.1
Saudi-Arabic	6	11.1	2.7
Qatar	4	7.4	1.8
Afghan	3	5.6	1.4
Kuwait	2	3.7	0.9
Iranian	2	3.7	0.9
Iraqi	1	1.9	0.5
Isreali	1	1.9	0.5
Azerbaijani	1	1.9	0.5
Libanesi	1	1.9	0.5
Total	54	100.0	24.7
Western and Central Europe			
French	5	29.4	2.3
Dutch	4	23.5	1.8
Swiss	4	23.5	1.8
British	3	17.6	1.4
Belgian	1	5.9	0.5
Total	17	100.0	7.8
Africa			
Lybian	7	46.7	3.2
Moroccan	2	13.3	0.9
Sudanese	2	13.3	0.9
Egyptian	1	6.7	0.5
Angolan	1	6.7	0.5
Eritrean	1	6.7	0.5
Congolese	1	6.7	0.5
Total	15	100.0	6.8
Asia			
Thai	2	28.6	0.9
Vietnamese	2	28.6	0.9
Filipino	1	14.3	0.5
Indonesian	1	14.3	0.5
Japanese	1	14.3	0.5
Total	7	100.0	3.2
North and South America			
American	6	85.7	2.7
Brazilian	1	14.3	0.5
Total	7	100.0	3.2

### Response to treatment

Differences in response to treatment of the foreign and the German patients’ groups were detectable (Table 4). One hundred thirty-five foreign patients (61.1%) achieved a CR compared to 146 German patients (66.7%) with CR. Eleven foreign patients (5.0%) achieved a PR in comparison to 19 Germans (8.7%) with PR. SD was seen in 24 foreign (11.0%) and 11 German patients (5.0%). PD was experienced by 26 foreign (11.9%) and by 21 German patients (9.6%). The response of 19 foreign (8,7%) and 18 German (8.2%) patients could not be assessed. Four matched pairs did not receive any treatment.

**Table 4:** Response to treatment. Patients with an unknown response or without any treatment irrespective the corresponding matched patients were excluded in the comparison of CR plus PR vs. SD plus PD

	Foreigners (*n* = 219)		German (*n* = 219)		Overall (*n* = 438)		chi-square
Response	n	%	n	%	n	%	
CR	135	61.1	146	66.7	281	64.2	0.273
PR	11	5.0	19	8.7	30	6.8	0.130
SD	24	11.0	11	5.0	35	8.0	0.022
PD	26	11.9	21	9.6	47	10.7	0.440
unknown	19	8.7	18	8.2	37	8.4	0.864
No therapy received	4	1.8	4	1.8	8	1.8	1.000
Total	219	100.0	219	100.0	438	100.0	
CR or PR	145	78.0	156	83.9	301	80.9	0.147
SD or PD	41	22.0	30	16.1	71	19.1	
Total	186	100.0	186	100.0	372	100.0	

The frequency of SD in foreign patients was significantly higher compared to the frequency of SD in the German patients group (chi square, *P* = 0.022).

Regarding the Overall Remission Rate (ORR) which compares CR and PR versus SD and PR, the following distribution was given: 145 foreign patients (78.0.%) and 156 German patients (83.9%) achieved a CR or PR. Fourty one foreign patients (22.0%) and 30 (16.1%) German patients achieved a SD or PD (chi-square, *P* = 0.147).

In a subgroup analysis including 324 patients with colorectal, urological and gynecological cancer, cancer of the stomach, thyroid gland, bronchus and lung, male genital tract, head and neck, esophagus, liver and biliary tracts, melanoma, sarcoma, leukemia, lymphoma and multiple myeloma (162 matched pairs), the soft tendency of a foreign patients’ worse response could be substantiated with a chi-square lower than 0.05. One hundred thirty-one foreign patients (80.9%) and 144 German patients (88.9%) achieved a CR or PR. Thirty one foreign patients (19.1%) and 18 (11.1%) German patients achieved a SD or PD (chi square, *P* = 0.044). However, this cohort contained 65 matched pairs with incomplete corresponding therapies. After excluding the matched pairs with incomplete correspondence in therapy, the differences in response disappeared (chi-square, *P* = 0.204).

### Survival analysis

The survival of both groups was compared by Kaplan-Meier analysis.

#### Overall survival

**Fig. 2:**
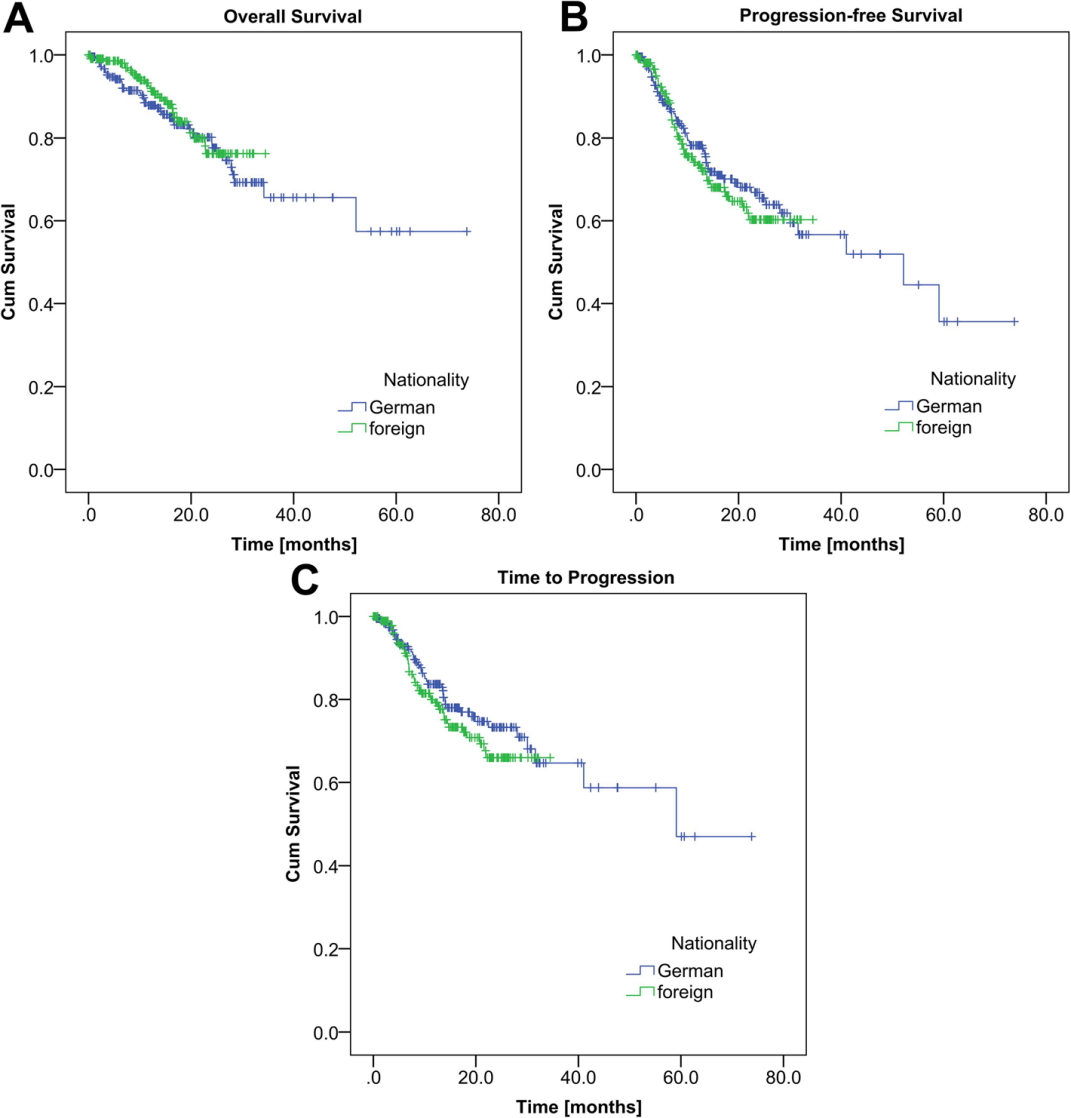
Kaplan-Meier analysis including all entities. **a** Overall survival of the German and foreign cancer patients (*n* = 438). Mean OS was 29.8 months for the foreign cohort versus 52.9 months for the German cohort (log rank, *P* = 0.477). **b** Progression-free survival of the German and foreign cancer patients (*n* = 434, 2 matched pairs had to be excluded due to insufficient clinical data). Mean PFS was 24.8 months for the foreign cohort versus 43.3 months for the German cohort (log rank, *P* = 0.522). **c** Time to progression of the German and foreign cancer patients (*n* = 398, 20 matched pairs had to be excluded due to insufficient clinical data). Mean TTP was 26.4 months for the foreign cohort versus 49.5 months for the German cohort (log rank, *P* = 0.295)

Mean OS was 29.8 months for the foreign patients’ group (*n* = 219) versus 52.8 months for the German patients’ group (n = 219) (Fig. 2a; log rank, *P* = 0.477). Twenty eight foreign and 41 German patients died during the time of the study.

#### Progression-free survival

Mean PFS was 24.8 months for the foreign patients’ group (*n* = 217) versus 43.3 months for the German patients (n = 217) (Fig. 2b; log rank, *P* = 0.522). 54 foreign (24.9%) and 44 German (20.3%) cancer patients experienced a disease progression or recurrence. 6 foreign and 19 German patients died without known disease progression before death.

#### Time to progression

Mean TTP was 26.4 months for the foreign patients’ cohort (*n* = 199) versus 49.5 months for the German patients’ cohort (*n* = 199) (Fig. 2c; log rank *P* = 0.295).

### Survival analysis of the different entities

Survival analysis was performed for the four most common entities including gynecological, colorectal and anal, urological and head and neck cancer.

#### Gynecological cancer

Mean OS for the foreign patients (*n* = 37) was 29.6 months versus 59.8 months for the German patients (*n* = 37) (log rank, *P* = 0.945). Mean PFS for the foreign patients was 28.2 months versus 53.7 months for the German patients (log rank, *P* = 0.968). Mean TTP for the foreign patients was 58.5 months versus 28.8 months for the German patients (log rank, *P* = 0.857). Mean follow-up time was 15.4 months for the foreign subgroup and 28.1 months for the German subgroup with gynecological cancer.

#### Colorectal and anal cancer

Mean OS for the foreign patients (*n* = 19) was 26.0 months versus 21.7 months for the German patients (*n* = 19) (log rank, *P* = 0.239). Mean PFS for the foreign patients was 14.0 months versus 17.3 months for the German patients (log rank, *P* = 0.335). Mean TTP for the foreign subgroup was 14.2 months versus 17.3 months for the German subgroup (log rank, *P* = 0.400). Mean follow-up time was 13.2 months for the foreign patients and 12.6 months for the German patients.

#### Urological cancer

Mean OS for the foreign patients (*n* = 18) was 31.0 months versus 26.2 months for the German patients (*n* = 18) (log rank, *P* = 0.619). Mean PFS for the foreign patients was 25.6 months versus 24.5 months for the German patients (log rank, *P* = 0.841). Mean TTP for the foreign patients was 28.1 months versus 27.0 months for the German patients (log rank, *P* = 0.688). Mean follow-up time was 15.6 months for the foreign patients and 15.2 months for the German patients with urological cancer.

#### Head and neck cancer

**Fig. 3:**
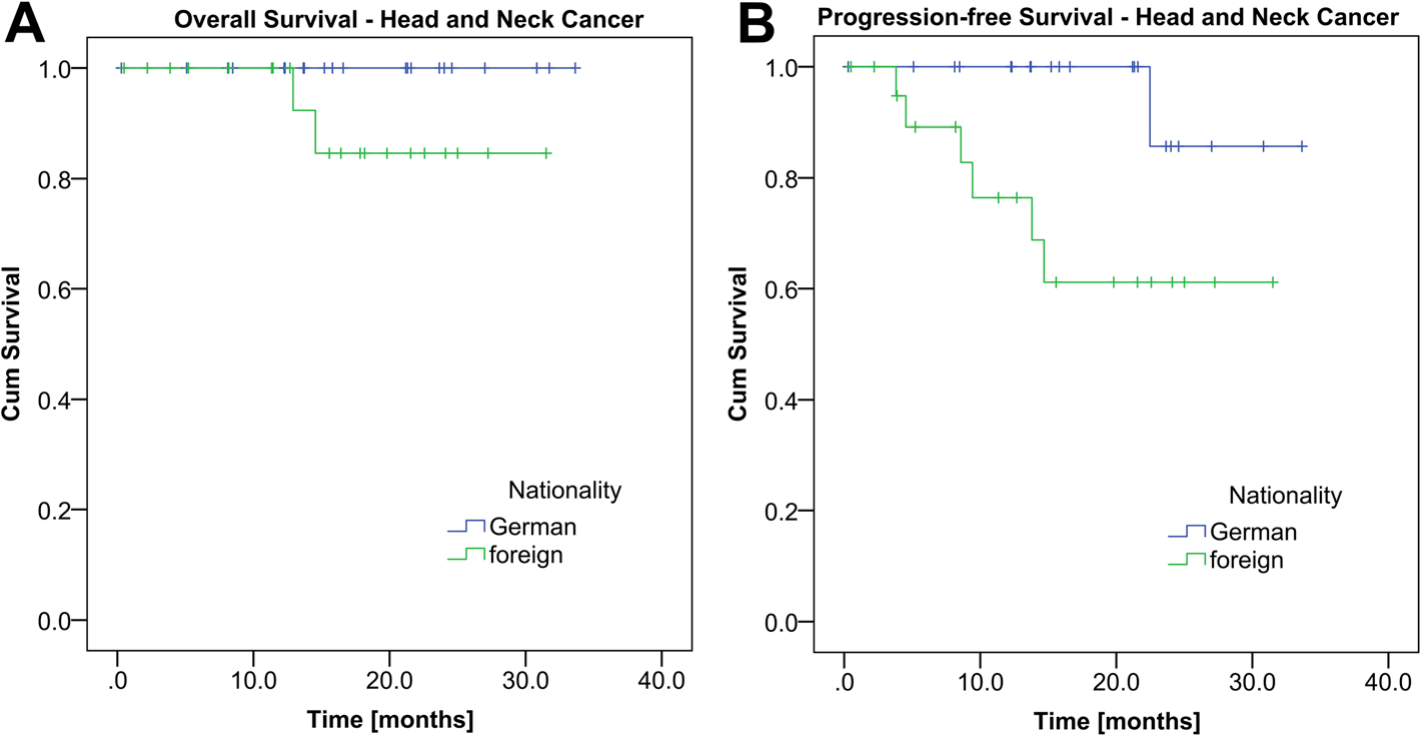
Kaplan Meier analysis including the patients with head and neck cancer. **a** Overall survival of the German and foreign patients with head and neck cancer (*n* = 42) (log rank, *P* = 0.135). **b** Progression-free survival of the German and foreign patients with head and neck cancer (*n* = 42). Mean PFS for the foreign cohort was significantly shorter with 23 months versus 32 months for the German cohort (log rank, *P* = 0.027)

During the time of the study, 3 foreign patients died while all German patients survived (Fig. 3a; log rank, *P* = 0.066). Mean PFS for all foreign patients (*n* = 21) with head and neck cancer was significantly lower with 23 months versus 32 months for the German patients (*n* = 21) (Fig. 3b; log rank, *P* = 0.027). As every patient of the two groups experienced a disease progression before death, the TTP was identical with the PFS. Mean follow-up time was 15.4 months for the foreign patients and 18.1 months for the German patients. As required, no significant differences within the matching parameters diagnosis (chi-square, *P* = 1.000), disease status (chi-square, *P =* 1.000), tumor stage (chi-square, *P =* 0.952), age (Students’ t, *P* = 0.127) and sex (chi-square, *P* = 0.432) existed. In 5 cases of the 21 matched pairs, differences in therapy could be found. In one case, the German patient received adjuvant chemotherapy after surgery and radiation whereas the matched foreign patient did not. The reverse constellation could be found in another matched pair. The patients of another matched pair differed from each other as the German patient received radiation after surgery in contrast to his foreign matching partner who did not. However, the reverse case existed as well. Two patients who were matched to each other differed in the type of chemotherapy they received. The German patient received Carboplatin because of insufficient renal function whereas the foreign patient got Cisplatin. As all differences except the last one were counterbalanced by each other and the other two patients both received a derivative of platinum, we decided to accept the differences in our further calculations.

The response rates of the foreign and the German patients’ group had the following distribution: 18 foreign (85.7%) and 19 German (90.5%) patients achieved a CR. One foreign (4.8%) and one German (4.8%) patient achieved a PR. PD was experienced by 2 foreign (9.5%) patients. SD was not found at all.

Regarding the ORR, analysis lead to the following results: CR or PR was achieved by 19 foreign (95.0%) and 20 German (100%) patients while SD or PD was experienced by one foreign (5.0%) and no German patient (chi-square, *P* = 0.311).

## Discussion

This matched pair analysis intended to reveal differences in the outcome of German and foreign cancer patients being treated at the CIO in the University Hospital of Bonn. We found no evidence of disparities in survival comparing the complete cohort of foreign cancer patients with the matched German cohort (*n* = 438). Interestingly, a subgroup analysis of 21 German and 21 foreign patients with head and neck cancer revealed a significantly shorter progression-free survival for the foreign patients (log rank, *P* = 0.027). In Germany, during the year 2014, 4.560 women and 12.660 men were diagnosed with a head and neck tumor. In 75% of these cases, tobacco use and alcohol could be seen as the main risk factors [8]. Other risk factors like infections with HPV and EBV are seen less often in Germany but gained more importance during the last years [9].

Our subgroup analysis of head and neck cancer patients has a small number and consequently limited power. However, similar results can be found in other studies:

Chen et Al observed that more aggressive oropharyngeal cancers occurred more frequently in a group of African American compared to a group of Non-Hispanic white Americans both living and being treated in the U.S.A. [10]. They included important prognostic factors like age, sex, alcohol and tobacco use, tumor stage and treatment in their study and hence avoided to create a matching bias. Their results suggest that there must be a biologically based racial disparity among oropharyngeal cancer patients explaining the poorer outcome of the African Americans. Unfortunately, we did not have sufficient data about alcohol and tobacco use. According to Chen et Al, this lack of information may explain many of the racial disparities reported for head and neck cancer survival in the literature. Hence, future plans should include data about alcohol and tobacco use to delineate the differences in outcome more accurately.

According to Arnold et Al, migrants are prone to cancers related to infections experienced in early life [7] and it is known that HPV and EBV infections are associated with the carcinogenesis of head and neck cancer [11, 12]. Correspondingly, a study of nasopharyngeal and hypopharyngeal carcinoma risk among immigrants in Sweden showed an increased risk for both entities in the cohort of immigrants revealing EBV to be the main environmental exposure influencing this risk [13].

The influences of EBV and HPV in survival are discussed controversially. Most studies associate HPV presence in head and neck cancer with a favorable prognosis [14–19] whereas others observe that this may not always be the case [20–22]. The same controversy can be found for the relationship between EBV infection and survival [11, 23]. According to Turunen et Al, the use of inappropriate laboratory EBV detection techniques may lead to a misunderstanding concerning the influence of EBV. They recommend the use of a highly sensitive in-situ-hybridization (ISH) of EBV encoded small RNAs (EBERs) to detect EBV in cancer cells instead of using a polymerase chain reaction (PCR) which mainly detects EBV DNA in lymphocytes. The presence of (EBV positive) lymphocytes in a tumor as a sign of immune response and favorable prognosis [24] should not be confounded with the presence of EBV in cancer cells. In their study, using ISH, EBV in head and neck cancer cells was associated with poor prognosis. Furthermore, a co-infection of EBV and HPV in head and neck cancer cells was associated with an even worse outcome.

Combining their results with the fact that migrants are prone to cancers related to infections experienced in early life [7], it can be hypothesized that the differences in the outcome of the German and foreign head and neck cancer patients’ cohorts are based on differences in carcinogenesis. A higher rate of EBV-infections and EBV/HPV-co-infections in the foreign patients’ cohort may explain the foreign patients’ poorer survival. Unfortunately, sufficient data about EPV and HPV infection status were not available for analysis. Further studies should focus on this hypothesis and follow-up should be extended for further years.

In our study, we observed a significantly higher rate of SD as status of response in the complete foreign patients’ cohort compared to the German cohort (chi-square, *P* = 0.022). This result is most likely associated with the fact that the foreign patients’ mean time of follow up is shorter than the German patients’ one. After a complete resection (R0), CR was chosen as status of response if the time of follow-up after the operation was longer than 1 month. If the time of follow-up was shorter than 1 month, SD was employed. As 6.4% of the foreign patients and only 3.7% of the German patients had a follow-up that lasted shorter than 1 month, it is reasonable to say that the higher rate of SD can be attributed to different lengths of follow-up.

We were also able to describe a significantly worse response to therapy for the foreign patients in a subgroup analysis including 324 patients with a large variety of cancers (chi square, *P* = 0.044). However, this cohort contained 65 matched pairs with incomplete corresponding therapies. As the differences in response disappeared after excluding the matched pairs with incomplete correspondence in therapy, the result can presumably be attributed to the differences in therapy. These findings should lead to further research delineating the reasons for the different treatment decisions more precisely. Especially the question if communication difficulties play a role in treatment decisions and enforcement should be tried to be answered. For 27.7% of the foreign patients included in the subgroup analysis, information about the necessity of an interpreter to communicate with the health-care team, were available. It is reasonable to assume that the number of foreign patients having communication difficulties is even higher, as fluency in everyday conversation may not be sufficient for conversations containing medical terminology [25]. Lee et Al observed that limited language proficiency is an immense handicap for Asian women with breast cancer in the U.S.A. hindering them from understanding medical information and making treatment decision [25]. Accordingly, Hyatt et Al detected an increased morbidity, mortality and psychological distress in migrant cancer patients in Australia, which they seem to be linked to language and communication difficulties as well as cultural-dependent differences in the understanding of health and illness and the health-care system [26]. Hence, it can be supposed that difficulties in communication may have influenced treatment decision in our cohort as well.

The cultural-dependent difficulties described above are also seen as an indicator for the existing lower participation of migrants in prevention programs compared to the German host population [27]. Not only in Germany, but also in other countries with widely accessible health care systems like Belgium, Italy and Spain, migrants’ access to preventive health programs and secondary cancer prevention programs is problematic and may lead to a late detection of cancer diseases, failure of attending follow-up consultation and lack of cancer awareness [28]. A longer time of follow-up may detect such possible aftereffects in our cohort.

There were several limitations to our study. First, our study is retrospective. Second, comorbidity was not assessed which most likely has an impact on overall survival. Third, our cohort represents a broad spectrum of time points in diagnosis and treatment, as otherwise an adequate matching would not have been possible. Fourth, we did not have data for and thus were unable to include socioeconomic status and the participation in prevention and aftercare programs. According to Jansen et Al, cancer patients from socioeconomically deprived regions have a worse survival than those living in affluent regions [29]. Socioeconomic deprivation is associated with advanced tumor stages at primary presentation and poor survival in a variety of cancers [5, 6, 29–31]. Hence, a more detailed, socioeconomic characterization of the foreign patients’ cohort is necessary to expose a potential social gradient in cancer survival. Fifth, we were not able to make a difference between foreigners and immigrants. Immigration can be defined as the process when a person moves his or her center of living over a socially meaningful and international distance [27]. In our study, as only non-German nationality was considered to be the selection criterion for the foreign patients’ cohort, e.g. naturalization might have obtained a possible immigrant status. Conversely, patients being born in Germany and living there since their birth, but not having a German nationality, were included in our study. In further studies, the foreign patients’ heterogeneity should be taken more into account.

## Conclusions

Despite the limitations mentioned above, we are encouraged that our findings will lead to further research evaluating the role of foreign nationality in the outcome of cancer patients in Germany. Availability of appropriate health care should be ensured for each patient irrespective of the social or cultural background or the presence of language barriers. The development of special programs teaching physicians skills how to deal with communication problems should be discussed, as well as programs for foreign patients considering their special needs and questions. Moreover, further studies may lead to a better understanding of carcinogenesis. In this context, particularly infection-related cancer entities like head and neck cancer should be investigated more detailed.

## Data Availability

The datasets used and analyzed during the current study are available from the corresponding author on reasonable request.

## Abbreviations

CIO: Center for Integrated Oncology
CR: Complete remission
EBERs: EBV encoded small RNAs
EBV: Epstein-Barr virus
HPV: Human papillomavirus
ISH: In-situ-hybridization
ORR: Overall remission rate
OS: Overall survival
PCR: Polymerase chain reaction
PD: Progressive disease
PFS: Progression-free survival
PR: Partial remission
RECIST criteria: Response evaluation criteria in solid tumors
SD: Stable disease
TTP: Time to progression
